# Identifying peer experts in online health forums

**DOI:** 10.1186/s12911-019-0782-3

**Published:** 2019-04-04

**Authors:** V.G.Vinod Vydiswaran, Manoj Reddy

**Affiliations:** 1 Department of Learning Health Sciences, University of Michigan, 300 N. Ingalls St, Ann Arbor, 48108 MI USA; 2 Department of Computer Science, University of California, Los Angeles, 404 Westwood Plaza, Los Angeles, 90095 CA USA

**Keywords:** Peer experts, Health forum analysis, Online health communities

## Abstract

**Background:**

Online health forums have become increasingly popular over the past several years. They provide members with a platform to network with peers and share information, experiential advice, and support. Among the members of health forums, we define “peer experts” as a set of lay users who have gained expertise on the particular health topic through personal experience, and who demonstrate credibility in responding to questions from other members. This paper aims to motivate the need to identify peer experts in health forums and study their characteristics.

**Methods:**

We analyze profiles and activity of members of a popular online health forum and characterize the interaction behavior of peer experts. We study the temporal patterns of comments posted by lay users and peer experts to uncover how peer expertise is developed. We further train a supervised classifier to identify peer experts based on their activity level, textual features, and temporal progression of posts.

**Result:**

A support vector machine classifier with radial basis function kernel was found to be the most suitable model among those studied. Features capturing the key semantic word classes and higher mean user activity were found to be most significant features.

**Conclusion:**

We define a new class of members of health forums called peer experts, and present preliminary, yet promising, approaches to distinguish peer experts from novice users. Identifying such peer expertise could potentially help improve the perceived reliability and trustworthiness of information in community health forums.

## Introduction

The digital revolution has led to tremendous growth in production and consumption of data online in numerous fields, including communication, shopping, travel, and health care. Internet users freely share their opinions and ratings on product review sites, interact with each other on social networking platforms, and ask and respond to questions on community forums. This shift in people-centered information exchange has shifted the task of generation of content solely from subject experts to a larger community of laypersons. For instance, there are over 5.6 million articles in English Wikipedia that are contributed by over 33 million volunteers [1]. The effect is even more prominent in specialized domains such as health and wellness, where online users access information related to their health and well-being through health portals and general-purpose search engines. According to a recent Pew Research survey [2], 87% of the US adults use the Internet and 72% of Internet users have looked for health information online. The same survey also reported that 77% of those online health seekers use a search engine to initiate their query, while only 13% of users initiate their search directly on a specialized health information website such as WebMD.

One of the prominent ways of accessing health information online is through discussion boards and community question-answering forums. These websites are generally organized as a list of threads, often grouped into related topics. In a previous work, Vydiswaran et al. [3] found that close to 50% of member communities in a health forum were focused on a specific disease or treatment, while another 16% were formed with the main intent of discussing pregnancy related concerns. In a typical health forum, a participant could issue a search query using keywords to identify relevant questions and articles of interest, and read through the responses and comments posted by other participants. Members could also post a new question to initiate new discussion threads and solicit responses from other members in the community. In response to posted questions, other members in the community would share their experiences and offer advice. Some health forums also include doctors and medical professionals who respond to user queries and participate in user-initiated discussion threads. Among the member participants, there is another set of users who, although not medically-trained, have extensive knowledge about particular diseases, treatments, or certain specific areas of health. They are prolific in responding to other users’ queries on those topics. We refer to such users as peer experts. Peer experts are capable of assisting other users by imparting factual knowledge regarding certain diseases and treatments or providing moral support and assisting in recovery. These users have the potential to significantly improve the health of other users by providing valuable advice and recommendation that might not be easy to find elsewhere.

In this paper, we formally characterize peer experts in health forums and develop methods to identify peer experts among the much larger set of member participants in a community health forum. Identifying peer expertise could help develop peer-to-peer recommendation systems to improve community-based support for chronic care management and in building e-medicine and e-health monitoring applications.

### Related work

There have been recent works that demonstrate the growing usage of health forums. Hoffman-Goetz et al. [4] have analyzed the content posted in response to queries about Type II Diabetes on an online health forum. They found that responses and recommendations provided were in high accordance with the clinical best practice guidelines. They argue that there exist such knowledgeable users, who we call peer experts, who have high health literacy skills and are interested in sharing this information. The work by Tanis [5] claims that the surge in the usage of health-related forums can also be attributed to various social factors, one of them being its affordance of anonymity. Patients who might feel stigmatized by their health condition are more comfortable participating in online discussion “anonymously” and maintain connections with similar patients.

The domain of community generated websites has also been explored widely. Adamic et al. [6] explored Yahoo! Answers, a popular question-answering website, and categorized different types of interactions and user behavior on the website. They proposed using user attributes and answer characteristics to predict whether a given answer to a question will be selected as the best answer or not.

There has been some relevant work done in the area of finding experts in online communities. Liu et al. [7] used information retrieval techniques by treating the query posted by a novice user as the query and the member profiles as the candidate documents. The retrieval techniques they used were language models such as the query likelihood models, relevance models, and cluster-based language models. The work of Pal and Konstan [8] introduced a new concept called ‘question selection bias’. They claimed that experts tend to answer only those questions that don’t already have good answers and showed that it was possible to find peer experts based on this selection bias. They focus on two datasets from other domains, namely the TurboTax community (personal finance) and StackOverflow (computer programming). Riahi et al. [9] tackle the same problem of finding experts on StackOverflow using topic modeling. They find that topic models perform much better than other retrieval techniques to find a set of best experts to answer a question. They were also able to show that Segmented Topic Model performed better than the Latent Dirichlet Model for this task. Two other papers by Jurczyk and Agichtein [10] and Zhang et al. [11] focus on using network connections and link analyses to predict experts in an online community. They use algorithms such as PageRank [12] and HITS [13] to find members with high influence in a network. The approaches followed by these authors have not been studied over health forums.

In this paper, we aim to study peer expertise in online health forums and how to identify them. Our approach differs from the previous ones in that we focus on text features to identify peer experts and understand how they evolve over time using temporal pattern analysis. This notion of using temporal patterns as features for machine learning has already been explored in other research works. Deushl et al. [14] have proposed using it to classify tremors based on tremor time series analysis. They found the waveform analysis to be highly informative to distinguish physiologic tremors in normal people from patients with Parkinson’s disease. Another work by Toshniwal and Joshi [15] used time weighted moments to compute the similarity between time series data. The main intuition behind using moments is that the centroid values within a given time interval is an effective way to represent the data trend that might be dense otherwise. We take a similar approach to summarize a user activity behavior and use central moments as features for the peer expert classification. As will be described in the next section, we compute the central moments of time series data that represents the activity level of each user and use it as features for our classification task.

## Methodology

### Data description

For this study, we used a dataset collected from a health forum website, MedHelp.com. MedHelp is one of the earliest and well-known online forums dedicated to supporting user-driven discussions on health or healthcare-related topics. As of February 2013, the website had over 12 million registered users who had collectively contributed over six million messages. The dataset includes all the posts on the website, along with the user profiles of all active registered users. The profiles include the groups that users have joined and a list of other users they are friends with. Table 1 summarizes the dataset fields extracted from the website. The dataset has been analyzed in previous works ([3, 16]), and the health forum itself has been studied in several other research works ([17–19]).

**Table 1:** Attributes of a user profile in the MedHelp dataset

Attribute	Description
About	A brief description about the user
Status	Collection of status updates over time
Best Answers	Number of posts that have been voted as the best answer
Interests	Topics that the user is interested in
Posts	Contains the actual text of posts by the user
Journals and notes	The user is able to jot down important notes that might be of interest to others
Communities	List of communities that the user is a member of
Friends	List of user’s friends

### Defining peer experts

The users of an online health forum can be characterized into the following four categories:

- Silent users and forum visitors
- Novice users
- Peer experts
- Physicians

**Silent users** are those who have a membership account on the forum platform, but are not active participants, i.e. they do not post any message, question, or comment on the platform. Similar to **forum visitors**, who are web users without a membership to the forum, silent users consume the information content already on the website to satisfy their information needs. However, unlike forum visitors, silent users may have additional access to restricted content available only to members of the website. A significant majority of the forum members fall under this category of users.

The second category of users, **novice users** are those users who are moderately active on the website. These users post a few questions and might participate in discussions on specific topics of interest. Their main motivation is to follow the latest developments with regards to a particular disease, condition, or treatment and contribute their viewpoints when possible. They have limited medical knowledge and may occasionally respond to informational questions from other users.

The third type of users, and the focus of this study, is **peer experts**. We define a peer expert as a member who, although lacking formal professional medical training, is able to assist other users in improving their health. Some of the roles played by peer experts include providing emotional support to users suffering from a disease or sharing valuable advice to diagnose a particular symptom. At times, peer experts can also act as a role model through their personal struggles and experience of overcoming an ailment or undergoing a particular treatment. In general, peer experts gain expertise by researching on a specific medical condition and becoming knowledgeable over a period of time. These peer experts also exhibit altruistic behavior in terms of devoting significant time and effort to share vital information that might be of potential use to other users. They do so by actively replying to user questions on the online health forum and initiating or being a part of communities that are of interest to them. They are generally highly motivated individuals passionate about their chosen topic of interest and expertise.

The fourth category of users are **physicians** and other medically-trained professionals. These constitute a relatively small fraction of users in most community-based health forums. Physicians typically identify themselves in their profiles or posts to signal credibility. In some health forums, physicians offer their credentials to be validated and are distinguished from other members through explicit tags or symbols. In our dataset, physicians were identified by the ‘Rod of Asclepius’ next to their unique names on their profile and on all of their posts.

Among the four types of users in an online forum, the silent users and physicians are easily identifiable based on either an absence of any recorded activity or presence of an explicit, identifiable tag that marks physician accounts. The rest of the paper focuses on identifying peer experts from novice users.

### Identifying peer expertise

**Fig. 1:**
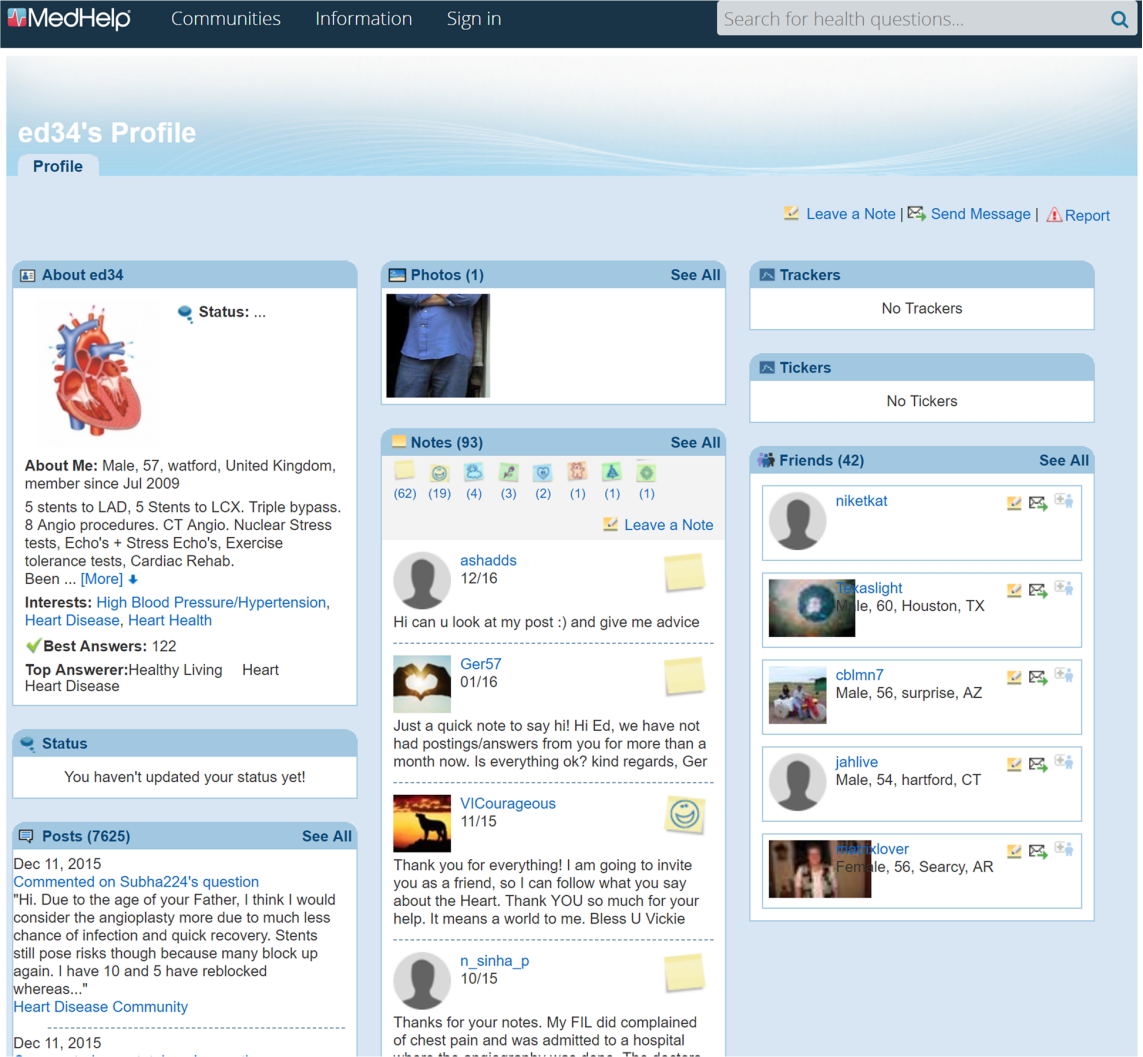
Profile example. Profile of a peer expert, “ed34”

Peer experts are primarily distinguished from novice users based on their skills, their activity level in their communities of interest, and the impact of their involvement through feedback from other members in the community. For example, Fig. 1 presents an example profile of a peer expert on MedHelp.org, an online health forum. The user (“ed34”) has a high activity level (7037 posts) and a high number of best answers (143) in the area of cardiovascular diseases. An example post by this user in the topic of bypass surgery is:

*“Why don’t you request a CT-Angio? This is different from standard Angiograms in that they are none[sic] invasive. I am wondering if something is wrong with the right side of your heart and maybe intervention can cure it.”*

Being a peer expert in the area of heart diseases, ed34 is able to assist another user on the website undergoing a particular treatment regimen, planning a surgery, or experiencing certain symptoms. This user is classified as a peer expert due to a high level of skill, knowledge, and motivation to help other users improve their health.

Identifying peer experts based on their skills is a difficult task. Instead, we characterize users based on their profiles and activity level. Each user is represented by a combination of the following features:

#### Activity-based features

These features aim to gauge the user activity level on the community health forum. Some of the features include the raw count of the total number of posts by the user and the number of days as a member on the health forum.

#### Textual features

The text from all posts authored by the user are collected and analyzed. Some of the textual features include the average length of posts, most frequent keywords used by the user, and the most common semantic classes represented in the posts.

The features related to semantic classes were computed using the Linguistic Inquiry and Word Count (LIWC) software [20]. LIWC utilizes lists of words and their linguistic variants for a number of complex semantic classes, such as emotive words for a range of emotions such as joy, anger, and sadness, words representing positive or negative sentiment, modality, etc. For a given text snippet, such as forum posts authored by a user, LIWC scores are computed based on the count of words that belong to these semantic word lists in the text snippet. For this study, LIWC scores corresponding to number of articles, cognitive words, and positive and negative emotion words were included in the feature representation. These scores help us understand the tone, emotion, and overall sentiment in posts authored by each user.

#### Network features

The network features act as an important proxy of activity and influence in the community where peer experts participate. To calculate the network features, we built a friendship graph using the friends’ list from user profiles. We then computed the PageRank of all users in the friendship network. Some of the other social network-based features include the number of friends and number of online health communities that the user is a member of.

### Time series analysis

**Fig. 2:**
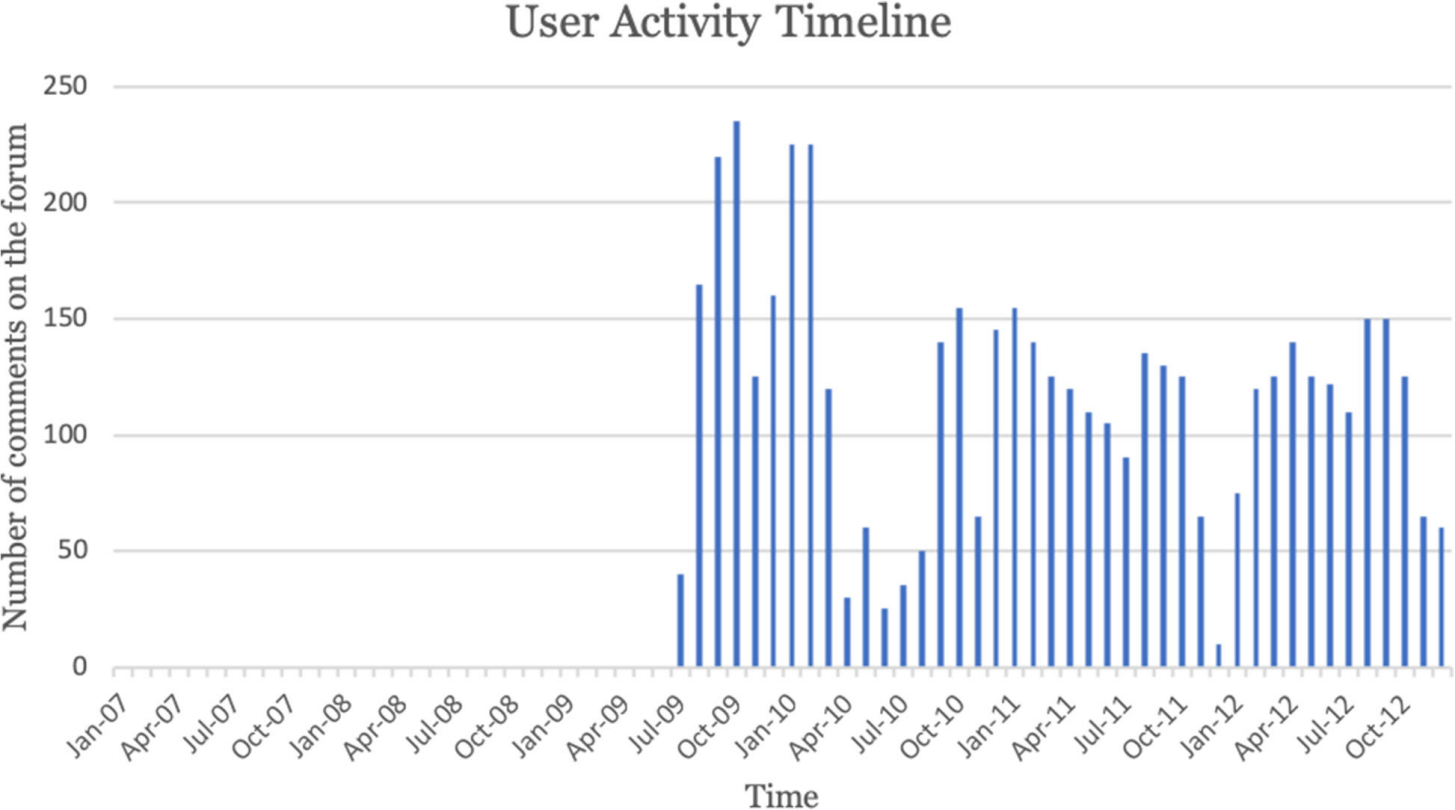
User activity timeline of a peer expert, “ed34”

To model evolution of user expertise over time, we computed the features described above as a discrete time series. Each time point represented one month of member activity on the health forum. The time series were shifted based on the joining dates for the members. For example, for a user who has been a member for six years, the time series would have 72 data points, one for each month of activity on the forum. As an example, Fig. 2 shows the activity timeline of the user “ed34”, the peer expert profiled in Fig. 1. The time series of the peer expert in Fig. 2 demonstrates the user’s activity pattern over time since he/she joined the website in July 2009. The activity timeline shows that this user is very active (averaging about 100 posts per month) and that the number of posts varies over time as the user gains experience and expertise within the platform.

#### Calculation of moments

Once the time series data for each user is collected, we summarize the overall activity by computing the moments of this discrete time signal. The following nine measures were computed: count, sum, mean, median, maximum value, minimum value, variance, skewness, and kurtosis. The first six parameters are standard statistical measures calculated on the set of the numbers representing the user activity data on the forum website. The last three are the second, third, and fourth central moments. The skewness measure represents the symmetry in the data, while kurtosis is a measure of whether the data distribution is “peaked” or “flat” relative to a normal distribution. Mathematically, they are calculated as:

$$\textrm{Skewness:} \qquad \frac{1}{N \times \sigma^{3}} \sum_{i} (y_{i}-\hat{y})^{3} $$

$$\textrm{Kurtosis:} \qquad \frac{1}{N \times \sigma^{4}} \sum_{i} (y_{i}-\hat{y})^{4} $$

where *y*_*i*_ is the individual data point, $\hat {y}$ is the mean, *N* is the number of data points and *σ* is the standard deviation. The moments obtained are then used as features for the classification task that is explained in the next section. The underlying assumption is that peer experts have similar behaviors in terms of user activity on the website which might be distinct from that of novice users.

### Training peer expert identifier

We modeled peer expert identification as a supervised classification task of distinguishing peer experts from novice users. We extracted all features types, viz. activity-based, textual, and network features described above, from the dataset fields summarized in Table 1. For this study, we considered peer experts to be users who had authored at least one post voted as the best answer. Once the features for all users were computed, we used the scikit-learn package [21] to train a logistic regression classifier and two support vector machine (SVM) classifiers with linear and radial basis function (RBF) kernels.

#### Handling class imbalance

To evaluate the performance of the trained classifiers and features on the task of identifying peer experts, we synthetically varied the ratio of peer experts to novice users in our training experiments. Starting from a balanced set with a near equal number of peer experts and novice users (ratio of 1:1, baseline accuracy = 0.5), we progressively added additional novice users to reduce the ratio of peer experts to novice users from 1:1 to 1:6. The results were then reported on the held-out test set.

In order to verify that the results of the classifiers are statistically significant, we ran multiple iterations of the same task by randomizing the data within each run. Specifically, we ran ten iterations of each of the classifier with different sets of data and randomizing the input order. We computed the t-values for every possible pair of classifier including the baseline to check for statistically significance. The *p*-value is then computed with a standard alpha value of 0.05.

## Results

### Data analysis

Table 2 summarizes the distribution of gender based on the information provided in the “About” section of the member profiles (see Table 1 for description). We observe that there are more females than males in the overall member population (61 to 39%), and that females are more likely to be users with at least one best answer, compared to males (72 to 28%).

**Table 2:** Overall gender statistics of MedHelp users

Gender	Number of users	Users with at least one best answer
Male	188,538 (38.9%)	396 (27.9%)
Female	295,851 (61.1%)	1,021 (72.1%)

On further analysis of the friendship network, we found that the users with at least one best answer were also well connected in terms of the number of friends. Peer experts represent a very small fraction of users. The ratio of peer experts to the total number of users is about 0.15%.

The ten most frequently mentioned topics based of interest mentioned in user profiles are summarized in Table 3. *Women’s health* and *Emotional health* were the most frequently mentioned topics. By analyzing the posts and features on the popular topics, we observed that the average length of posts by peer experts is often longer than that of novice users.

**Table 3:** Top ten topics of interest mentioned in user profiles

Rank	Topic	Frequency
1	Women’s health	200
2	Emotional health	173
3	Pregnancy	152
4	Pain	139
5	Sexual health	138
6	Allergies	129
6	Weight management	129
8	Depression	127
9	Anxiety	124
10	Nutrition	109

### Evaluation of classification models

**Fig. 3:**
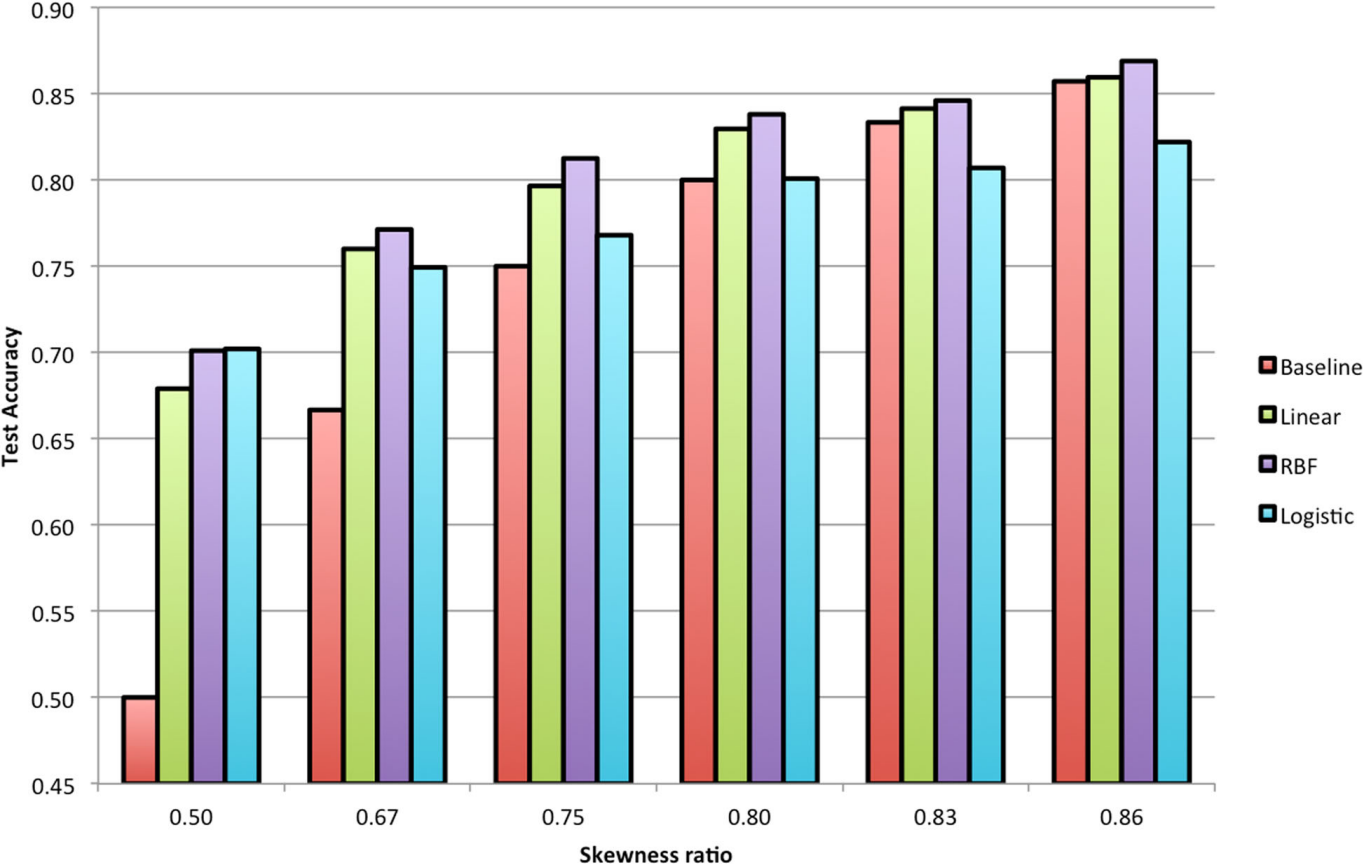
Classification accuracy on the test data

Figure 3 shows the results of the baseline model and the three trained classifiers, as we varied the skewness ratio of our training and test datasets. We find that when the training data is less imbalanced, all classifiers perform at least as well as, if not better than, the baseline model. However, when the class imbalance is high, the logistic classifier performs worse than the baseline model. The performance of the SVM classifier with RBF kernel was the best among all the classifiers across all skewness ratios. Statistical significance analysis shows that while all classification models are better than the baseline model at lower skewness ratios, with SVM classifier with RBF kernel significant at *p*<0.001 level. SVM classifier with RBF kernel remains significantly better than the baseline model at *p*<0.05 level for all skewness ratios. The difference is performance due to kernel variation in SVM classifiers was not found to be statistically significant.

### Feature analysis

On further analyses of the results from the logistic regression classifier, we found that the first twelve features corresponding to the highest (absolute) feature weights correspond to the LIWC features obtained using the text analysis. The most important feature from the time series is the mean of user activity per month. This value corresponds to the number of posts averaged across the user activity timeline. This seems intuitive since peer experts tend to be more active and respond to more posts than novice users. The most important activity-based feature was the binary feature of whether or not the total number of posts by a user is greater than or equal to 100. Hence, it implies that high user activity is a good indicator of being a peer expert. The number of friends was also an important factor but was not among the top fifteen features when ranked according to feature weights.

## Discussion

This study is an initial attempt to identify peer experts that could augment the professional healthcare services with emotional and information support in online health communities. Peer experts were also able to explain concepts better. For example, in a question posted on a forum on Diabetes about potential causes for fluctuating sugar levels, a response by a Diabetes peer expert was as follows:

*“It is impossible for us to evaluate you over the Internet. I suggest you get referred to an Endocrinologist – a doctor trained in diabetes care and treatment – for further testing. High levels after a meal then diving to lows 3–4 h later could possibly be reactive hypoglycemia. Only a thorough medical evaluation and testing can reveal the real cause. Good luck”*

This response demonstrates how peer experts could be a great resource to the community because of their topical expertise and willingness to share their knowledge and improve the overall health of the community. As community question answering health forums get more popular among patients and caregivers, peer experts could help moderate such community forums, resolve the more contentious discussions, and perhaps help in flagging misinformation in forum posts. Hence, identifying such peer expertise could potentially help improve the perceived reliability and trustworthiness of information in community health forums. Online health forum portals could be redesigned to highlight contributions of peer experts to improve the trustworthiness of information. Indeed, just as the ‘Rod of Asclepius’ identifies physicians, appropriate symbolism could be used to identify peer experts.

Additional studies are needed to further characterize peer experts in terms of their behavior and ways to nurture them. In particular, we plan to further study the progression of users from novice users to peer experts on online health forums and study how to improve the quality and trustworthiness of medical information on such community portals.

## Conclusion

In this study, we defined peer experts in an online health forum and presented techniques to identify peer experts in an online community. The features were collected based on the activity level, textual characteristics, and temporal progression of users as they gain expertise in the topic. We showed that these features are indeed helpful in training classifiers that help distinguish peer experts from novice users from highly skewed datasets.
